# Cyclophosphamide Induces the Ferroptosis of Tumor Cells Through Heme Oxygenase-1

**DOI:** 10.3389/fphar.2022.839464

**Published:** 2022-02-21

**Authors:** Hezhan Shi, Bo Hou, Huifeng Li, Hui Zhou, Bin Du

**Affiliations:** ^1^ Department of Pathology, Shanghai First Maternity and Infant Hospital, School of Medicine, Tongji University, Shanghai, China; ^2^ Department of Pathology, School of Medicine, Jinan University, Guangzhou, China; ^3^ Department of Pathology, The Third Affiliated Hospital, Sun Yat-Sen University, Guangzhou, China; ^4^ Department of Nasopharyngeal Carcinoma, Sun Yat-sen University Cancer Center, Guangzhou, China; ^5^ Department of Chemistry, Jinan University, Guangzhou, China

**Keywords:** cyclophosphamide, ferroptosis, heme oxygenase-1, nuclear factor E2 related factor 2, RNA sequencing

## Abstract

Ferroptosis has been implicated in the therapeutic responses of various types of tumors. Cyclophosphamide (CTX), one of the most successful antitumor agents, is widely used to treat both hematopoietic and solid tumors. In this study, we revealed the ferroptosis pathway targeted by CTX treatment in tumor cells and clarified its mechanisms. Cell viability was remarkably suppressed by CTX, accompanied by the accumulation of intracellular iron and reactive oxygen species (ROS), reduced glutathione levels, deformed mitochondria and a loss of the mitochondrial membrane potential. These effects were impeded by the ferroptosis inhibitors ferrostatin-1 (Fer1) and deferoxamine (DFO). Moreover, CTX treatment obviously upregulated nuclear factor E2 related factor 2 (NRF2) and heme oxygenase-1 (HMOX-1) expression. Additionally, the HMOX-1 inducer Hemin notably enhanced CTX-mediated tumor inhibition *in vitro* and *in vivo* through a mechanism that involved interfering with the ferroptosis process. Therefore, our findings indicated ferroptosis induction by CTX through the activation of the NRF2/HMOX-1 pathway, which might provide a potential strategy for tumor chemotherapy.

## 1 Introduction

Cyclophosphamide (CTX) is a commonly utilized broad-spectrum anticancer drug in clinical chemotherapy. The pharmacological mechanism of CTX has been explored in numerous studies, and it correlates with oxidative stress, inflammation, and apoptosis (Emadi et al., 2009). To date, one of the key challenges in cancer treatment is how to effectively kill cancer cells and minimize resistance to chemotherapy, which may limit the effectiveness of cancer therapies.

Unlike other types of programmed cell death (PCD), such as necrosis or apoptosis, ferroptosis, a newly discovered form of regulated cell death, depends on intracellular iron accumulation and subsequent lipid peroxidation and is related to factors such as glutathione (GSH) (Dixon et al., 2012; Yan et al., 2021). Ferroptotic death is morphologically, biochemically, and genetically distinct from apoptosis, various forms of necrosis, and autophagy (Friedmann Angeli et al., 2019). In addition to the induction of tissue injury and protective effects on various diseases, the activation of ferroptosis may also be responsible for the remarkable anticancer activity of target drugs such as sulfasalazine, sorafenib, and artemisinin (Chen et al., 2021). In addition, classic chemotherapeutic agents such as cisplatin have also been shown to induce ferroptosis in cancer cells (Dixon et al., 2012; Guo et al., 2018). However, few studies have deciphered the mechanisms connecting CTX and ferroptosis.

Heme oxygenase 1 (HMOX-1) is the rate-limiting enzyme in heme degradation, and upregulation of HMOX-1 increases ferritin synthesis, leading to a change in the intracellular iron distribution (Otterbein et al., 2003). As a stress response protein and a critical mediator of cellular homeostasis, the most important mechanism of HMOX-1 activation is mediated by nuclear factor erythroid 2-related factor 2 (NRF2). In cells under oxidative stress, free NRF2 translocates to the nucleus, where it drives the transcription of genes that contain an antioxidant response element (ARE) to activate its downstream antioxidant genes, such as HMOX-1 (Biswas et al., 2014). Therefore, activation of the NRF2 pathway, particularly with HMOX-1 induction, may participate in iron-dependent oxidative cell death by disturbing the balance of iron metabolism.

In this study, we first highlight that CTX is a new type of regulator of ferroptosis. By exploring the characteristics of ferroptosis in cancer cells and a tumor-bearing model *in vivo*, we revealed that ferroptosis was the relevant mechanism underlying CTX-triggered cell death. The identification of the mechanism underlying CTX-induced ferroptosis will provide the opportunity to optimize traditional chemotherapies and develop more strategies utilizing rational therapeutic regimens to improve clinical outcomes.

## 2 Materials and Methods

### 2.1 Antibodies and Reagents

The antibodies used in this study included NRF2 (16396-1-AP), Histone H3 (17168-1-AP), SLC7A11 (26864-1-AP), HMOX-1 (10701-1-AP), and GAPDH (16396-1-AP) (ProteinTech Group, IL, United States). 4-Hydroperoxy cyclophosphamide (4HC) was purchased from TCR (39800-16-3, Santa Cruz Biotechnology, TA, United States). Cyclophosphamide (CTX), Hemin, zinc protoporphyrin (ZNPP), ferrostatin-1 (Fer1), and deferoxamine mesylate salt (DFO) were purchased from Sigma–Aldrich (Sigma–Aldrich, MO, United States).

### 2.2 Cell Culture and Treatment

The murine glioblastoma cell lines (GL261, CT-2A, and KR-158) and murine breast cancer cell line (4T1) were kindly provided by Cell Bank, Chinese Academy of Sciences (Shanghai, China). Cells were maintained in Dulbecco’s modified Eagle’s medium (DMEM) supplemented with 10% fetal bovine serum and 1% penicillin/streptomycin (Gibco, Massachusetts, United States) in a humidified incubator at 37 °C with 5% CO_2_. Cells grown on 60 mm culture dishes (Corning, New York, NY, United States) were stimulated with or without different concentrations of 4HC (from 5 to 160 μM)for various times (from 0.5 to 48 h). Fer1 or DFO were added 1 h before 4HC supplementation. After treatment, cells were collected and used for subsequent experiments.

### 2.3 RNA Sequencing

RNA sequencing was performed by LC Sciences (Hangzhou, China). Briefly, total RNA was extracted from samples using TRIzol (Thermo Fisher Scientific, MA, United States) according to the manufacturer’s protocol. High-quality RNA was used to construct sequencing libraries and analyze the enrichment of differentially expressed genes (DEGs). The number of gene counts in each sample was normalized to the base mean value, and the *p* value and fold change (FC) of the difference in each comparison were calculated. The DEGs were screened based on *p* < 0.05 and FC > 2. Kyoto Encyclopedia of Genes and Genomes (KEGG) was used to perform the pathway enrichment analysis of the differentially expressed genes.

### 2.4 Cell Proliferation Assay

The CCK-8 assay was performed using a Cell Counting Kit-8 (CCK-8) (Bimake, TX, United States) according to the manufacturer’s protocol to assess cell proliferation. Cells were seeded in 96-well plates at a density of 5,000 cells per well. Cells were treated with a series of 4HC concentrations (from 0 to 160 μM) for various times (from 0 to 48 h) in the assay. Then, 100 μL of CCK-8 solution were added to each well and incubated at 37 °C for 1 h. Next, the absorbance of each well was measured at 450 nm.

### 2.5 Lactate Dehydrogenase Measurement

The amount of LDH released in cell culture supernatants was measured using the LDH Cytotoxicity Assay Kit (Beyotime Biotech, Nanjing, China) according to the manufacturer’s instructions. A total of 5,000 cells/well were treated with 4HC as described above in medium containing 1% FBS (Gibco, MA, United States). Sixty microliters of the reaction solution were added to each well and incubated in the dark at room temperature for 30 min. Then, the amount of LDH released was measured by detecting the absorbance at a wavelength of 490 nm, and cytotoxicity was calculated using the following equation: cytotoxicity (%) = (LDH released in the sample–background)/(total LDH release - background)  × 100.

### 2.6 Glutathione Measurement

GSH levels were measured using a GSH assay kit (Beyotime Biotech, Nanjing, China) according to the manufacturer’s instructions. Cells or lysed tissue were incubated with Protein Removal Buffer. The GSH concentration was measured in the supernatant collected from each well by detecting the absorbance at 412 nm. A standard curve was generated and used to determine GSH levels in the specimens.

### 2.7 Determination of the Iron Concentration Using ICP–MS

Cells or lysed tissue were washed twice and digested with a 3:1 ratio of trace element grade nitric acid:HCl for 20 min at 200 °C. Iron concentrations were analyzed using ICP–MS (inductively coupled plasma–mass spectrometry, PerkinElmer, MA, United States) according to the manufacturer’s instructions.

### 2.8 Determination of Reactive Oxygen Species Generation

Intracellular ROS levels were measured with the fluorescent probe 2′,7′-dichlorofluorescin diacetate (DCFH) (Solarbio, Beijing, China). As a cell-permeable nonfluorescent probe, DCFH is deesterified intracellularly and transformed into the highly fluorescent molecule 2′,7′-dichlorofluorescin (DCF) in the presence of intracellular ROS. Cells were incubated with 5 μM DCFH for 60 min at 37 °C. After the incubation, cells were observed using a Leica microscope system (Leica, Wetzlar, Germany) at an excitation wavelength of 488 nm and an emission wavelength of 525 nm or directly collected before the immediate detection of the fluorescence intensity of DCF using flow cytometry (BD Biosciences, CA, United States).

### 2.9 Flow Cytometry Analysis

The cells were digested and resuspended in PBS, and the fluorescence intensity was measured using flow cytometry (BD Biosciences, CA, United States). The MFI (mean fluorescence intensity) was calculated and displayed as a histogram using the FlowJo program (Tree Star, OR, United States).

### 2.10 Mitochondrial Membrane Potential Assay

The mitochondrial membrane potential was assayed using JC-1 according to the manufacturer’s instructions (Beyotime Biotech, Nanjing, China). Briefly, cells were cultured, treated with staining buffer for JC-1 at 37 °C for 20 min, and monitored using flow cytometry (BD Biosciences, CA, United States). The excitation wavelength of JC-1 is 488 nm, and the approximate emission wavelengths of the monomeric and J-aggregate forms are 529 and 590 nm, respectively.

### 2.11 RNA Isolation and Real-Time PCR

Total RNA was extracted from cultured cells or tissues with TRIzol (Thermo Fisher Scientific, MA, United States) according to the manufacturer’s instructions. After quantifying the concentrations and purities of RNA, 1 μg of RNA was reverse transcribed into cDNAs with an RT reagent Kit with gDNA Eraser (Takara, Dalian, China). Subsequently, cDNA templates from the samples were amplified with TB Green (Takara, Dalian, China). Quantitative PCR was performed using a C1000 Touch Thermocycler CFX96 Real-Time System (Bio-Rad, CA, United States). The relative expression of the target genes was standardized and determined using the 2^−ΔΔ^ CT method. All primers used for RT–qPCR are listed as follows:

18s-F: CGC​CGC​TAG​AGG​TGA​AAT​TC
18s-R: CCA​GTC​GGC​ATC​GTT​TAT​GG
Nrf2-F: AGC​AGG​CTA​TCT​CCT​AGT​TCT​C
Nrf2-R: AGA​TCT​ATG​TCT​TGC​CTC​CAA​AG
Slc7a11-F: TGC​TGG​CTT​TTG​TTC​GAG​TCT
Slc7a11-R: GCA​GTA​GCT​CCA​GGG​CGT​A
Fth1-F: CAC​TTG​GAA​AAG​AGT​GTG​AAT​CAG
Fth1-R: CGT​CTC​AAT​GAA​GTC​ACA​TAA​GTG
Hmox-1-F: TCA​CAG​ATG​GCG​TCA​CTT​C
Hmox-1-R: GTG​TCT​GGG​ATG​AGC​TAG​TG
Ptgs2-F: CTG​CGC​CTT​TTC​AAG​GAT​GG
Ptgs2-R: GGG​GAT​ACA​CCT​CTC​CAC​CA

### 2.12 Transmission Electron Microscopy

Cells were washed twice, fixed with 1% glutaraldehyde and postfixed with 2% osmium tetroxide. Following dehydration with a graded series of ethanol solutions, the samples were embedded in epon resin and cut into ultrathin sections using a microtome. The sections were then counterstained with saturated uranyl acetate and lead citrate before being examined with a transmission electron microscope (HITACHI, Japan). The morphological characteristics of mitochondria were observed in randomly selected cytoplasmic fields of view from each group.

### 2.13 Western Blot

After treatment, cells were washed with PBS, scraped for collection, gently suspended in extraction buffer with PMSF and incubated on ice for 10 min. The cell suspension was vigorously vortexed for 5 s to lyse the cells and release the cytoplasmic contents. The cellular mixtures were then centrifuged at 12,000-16,000 g for 5 min at 4 °C to separate the cytoplasmic components (supernatant) from the nuclear fraction (pellet). For the purification of nuclear proteins, the nuclear pellets were washed with hypotonic buffer three times and suspended in PBS containing proteinase inhibitors and phosphatase inhibitors. The suspension was further sonicated and centrifuged at 12,000 g for 10 min at 4 °C. The isolated fractions were stored at −80 °C until further analysis. The concentration of the protein lysate was measured using a BCA protein assay reagent kit (Thermo Pierce, MA, United States) and used for western blotting. The following primary antibodies were used at the indicated dilutions: 1:1,000 for NRF2, Histone H3, SLC7A11, HMOX-1, and GAPDH.

### 2.14 Transfection of Small Interfering RNAs/Gene Silencing With shRNA

The siRNAs targeting Hmox-1 were synthesized by EgyptBio (Egypt Biotechnology, Guangzhou, China). The shRNA segments for Nrf2 were donated by Professor Xuesong Yang from the Department of Histology Embryology, Jinan University. After reaching 80–90% confluence, cells were transfected with siRNAs or the specific shRNA using Lipofectamine 2000 Transfection Reagent (Thermo Fisher, MA, United States) in Opti-MEM (Thermo Fisher, MA, United States) according to the manufacturer’s instructions. After 6–8 h of incubation at 37 °C, the medium was replaced with complete medium. Cells were prepared for tests and harvested after 48 h. The sequences of siRNAs/shRNAs used for RNA interference are listed as follows:

Hmox-1-F: GGG​UCA​GGU​GUC​CAG​AGA​A
Hmox-1-R: UUC​UCU​GGA​CAC​CUG​ACC​C
NC F: UUC​UCC​GAA​CGU​GUC​ACG​U
NC R: ACG​UGA​CAC​GUU​CGG​AGA​A
Nrf2-F:
  GGG​CAA​GAT​ATA​GAC​CTT​GGT​CAA​GAG​CCA​AGG​TCT​ATA​TCT​TGC​CTT​TTT​TGA
Nrf2-R:
  GCA​GTC​TTC​ATT​TCT​GCT​AAT​CAA​GAG​TTA​GCA​GAA​ATG​AAG​ACT​GTT​TTT​TGA

### 2.15 Immunofluorescence Staining

Cells on glass slides were fixed with 4% paraformaldehyde for 15 min at room temperature. After two rinses with PBS, cells were permeabilized with 0.1% Triton (Sinopharm, Shanghai, China) for 5 min, incubated with a primary antibody against NRF2 at 4 °C overnight and secondary antibody at room temperature for 1 h, and counterstained with DAPI. Images were captured using a Leica microscope system (Leica, Wetzlar, Germany).

### 2.16 Animal Studies

Six-week-old female Balb/c mice were purchased from Huafukang Co. (Hfkbio, Beijing, China) and maintained under specific pathogen-free (SPF) conditions. The animal experiment was performed under the supervision of the Jinan University Animal Ethics Committee in accordance with the Animal Research: Reporting of *In Vivo* Experiments (ARRIVE) guidelines (approval no. 20180604–02). A total of 1 × 10^6^ 4T1 cells was injected into the mammary fat pad of each mouse. Once the tumor size reached ∼200 mm^3^, mice were randomly allocated to six different groups as follows (6-7 mice per group): 1) untreated (UT) group (PBS); 2) Fer1 treatment group (4 mg/kg); 3) Hemin treatment group; 4) CTX treatment group; 5) CTX + Fer1 treatment group; and 6) CTX + Hemin treatment group. CTX (140 mg/kg, i. p.) was administered every 6 days (Days 0, 6, 12 and 18) after Fer1 (4 mg/kg, i. p.), Hemin (20 mg/kg, i. p.) or the equivalent vehicle was administered 1 day in advance.

During the treatment period, the tumor size and body weights of the 4T1-bearing mice were monitored every other day. The tumor volume of each mouse was measured using the following equation: tumor volume (mm^3^) = 1/2 length (mm)  × width (mm)^2^. Then, the mice were sacrificed by cervical dislocation on Days 12 and 18 after the first administration of CTX. Tumor masses were harvested for further investigations.

### 2.17 Statistical Analysis

The experiments were all independently repeated at least three times. Statistical analysis were conducted using GraphPad Prism software (GraphPad, CA, United States). Significant differences between the groups were analyzed using one-way ANOVA, followed by Student-Newman-Keuls test or Student’s *t*-test; *p* < 0.05 was considered to indicate a statistically significant difference.

## 3 Results

### 3.1 Transcriptomic Analysis Revealed the Ferroptosis Pathway in CTX-Treated Tumor Cells

**FIGURE 1:**
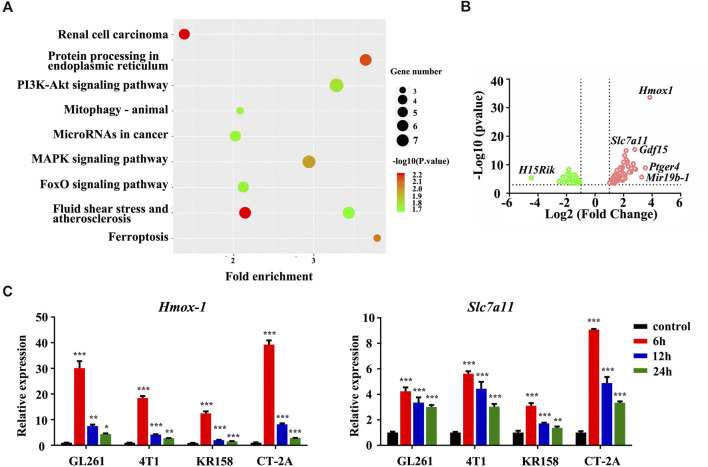
The ferroptosis pathway was involved in 4HC-treated tumor cells. **(A)** Dot plots show the enriched terms identified in the KEGG analysis of differentially expressed genes detected in 4HC-treated GL261 cells compared with vehicle-treated cells, and the ferroptosis pathway was altered. **(B)** Volcano plot showing the upregulated and downregulated genes in response to 4HC treatment measured using RNA-seq. Solid symbols indicate genes in which the differential expression was statistically significant. Red, upregulated genes; green, downregulated genes. **(C)** Relative expression of the Hmox-1 and Slc7a11 mRNAs in different cell lines treated with 4HC (20 μM) for 6, 12 and 24 h. Data are presented as the means ± SD from more than three independent experiments; *p < 0.05, **p < 0.01, and ****p < 0.001 (one-way ANOVA analysis).

We first performed RNA sequencing in GL261 murine glioblastoma cells treated with 4-hydroxycyclophosphamide (4HC), the pharmacologically active moiety of CTX, to systematically investigate the biological processes underlying the therapeutic effect of CTX. Differentially expressed genes were identified based on a *p* value <0.05 and fold change >2. Among differentially expressed genes, Hmox-1 was one of the most significantly upregulated genes, and the cystine/glutamate antiporter solute carrier family 7 membrane 11 (Slc7a11) gene was slightly upregulated (Figure 1B). The KEGG analysis based on RNA-seq data also revealed enriched pathways related to ferroptosis, suggesting that iron homeostasis was among the pathways that were remarkably altered in 4HC-treated cells (Figure 1A). In addition, some other enriched pathways such as protein processing in endoplasmic reticulum or the mitogen-activated protein kinases (MAPKs) were also shown. Consistently, the real-time PCR analysis confirmed that 4HC treatment led to a significant increase in Hmox-1 and Slc7a11 mRNA levels at an early stage (within 6 h) in the murine glioblastoma cell lines GL261, CT-2A, and KR-158 and the 4T1 murine breast cancer (Figure 1C). Therefore, the ferroptosis pathway tended to be altered by CTX in tumor cells, which at least partially involved HMOX-1 regulation.

### 3.2 Ferroptosis Inhibitors Reversed CTX-Induced Cytotoxicity and Oxidative Stress in Tumor Cells

**FIGURE 2:**
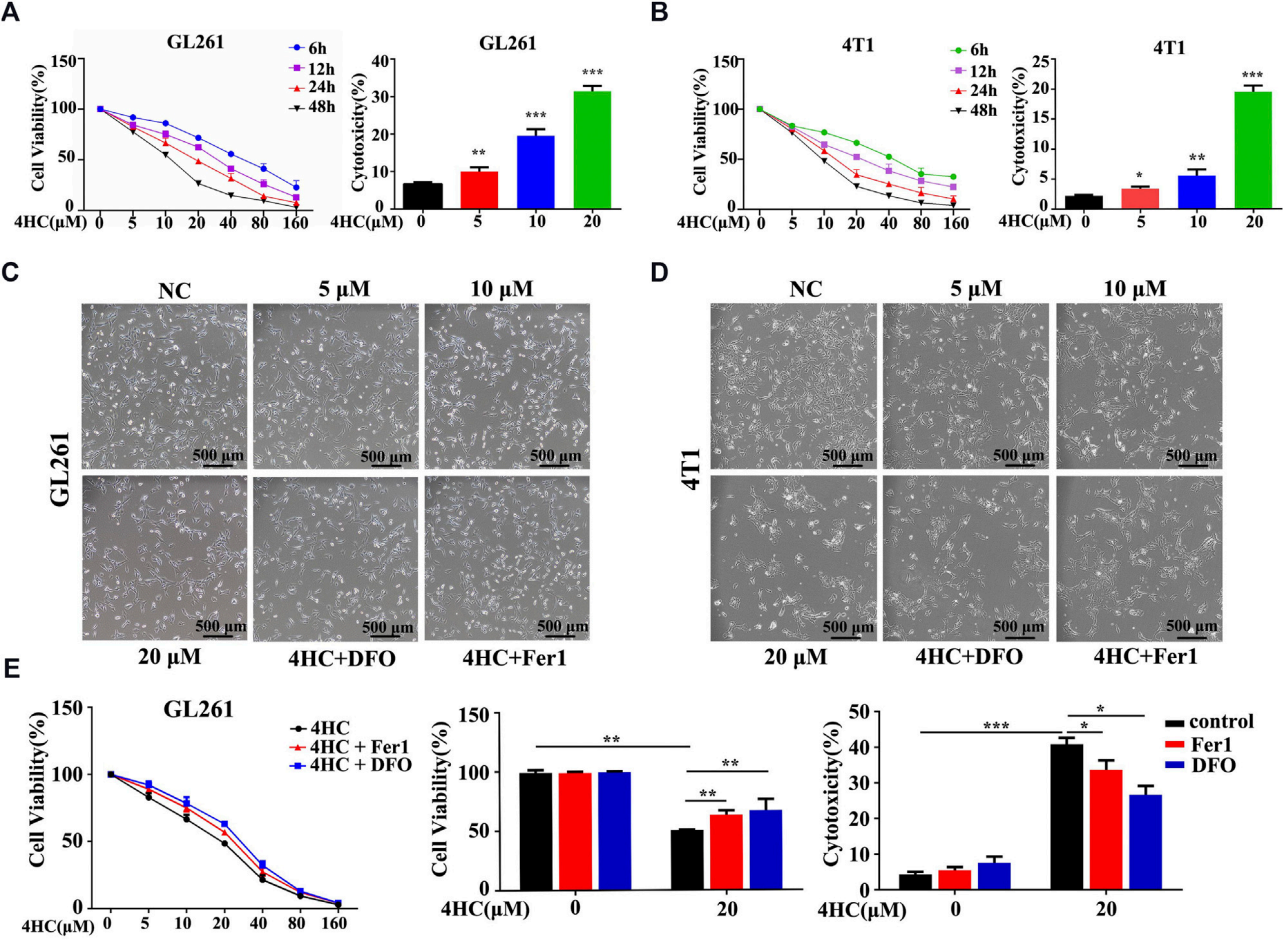
Ferroptosis inhibitors reversed 4HC-induced cytotoxicity **(A–B)** GL261 and 4T1 cell lines were treated with different concentrations of 4HC (0–160 μM) for 6, 12, 24 or 48 h **(C–D)** Cell morphology was photographed under an inverted microscope, and representative images from experiments with similar results are shown (scale bar = 500 μm) **(E)** GL261 cell line was preincubated with Fer1 (10 μM) or DFO (400 μM) for 1 h followed by 4HC (0–160 μM) for 24 h. The values of cell viability and cytotoxicity are presented as the means ± SD from more than three independent experiments.*p < 0.05, **p < 0.01, and ***p < 0.001 (one-way ANOVA analysis) for the indicated comparisons.

**FIGURE 3:**
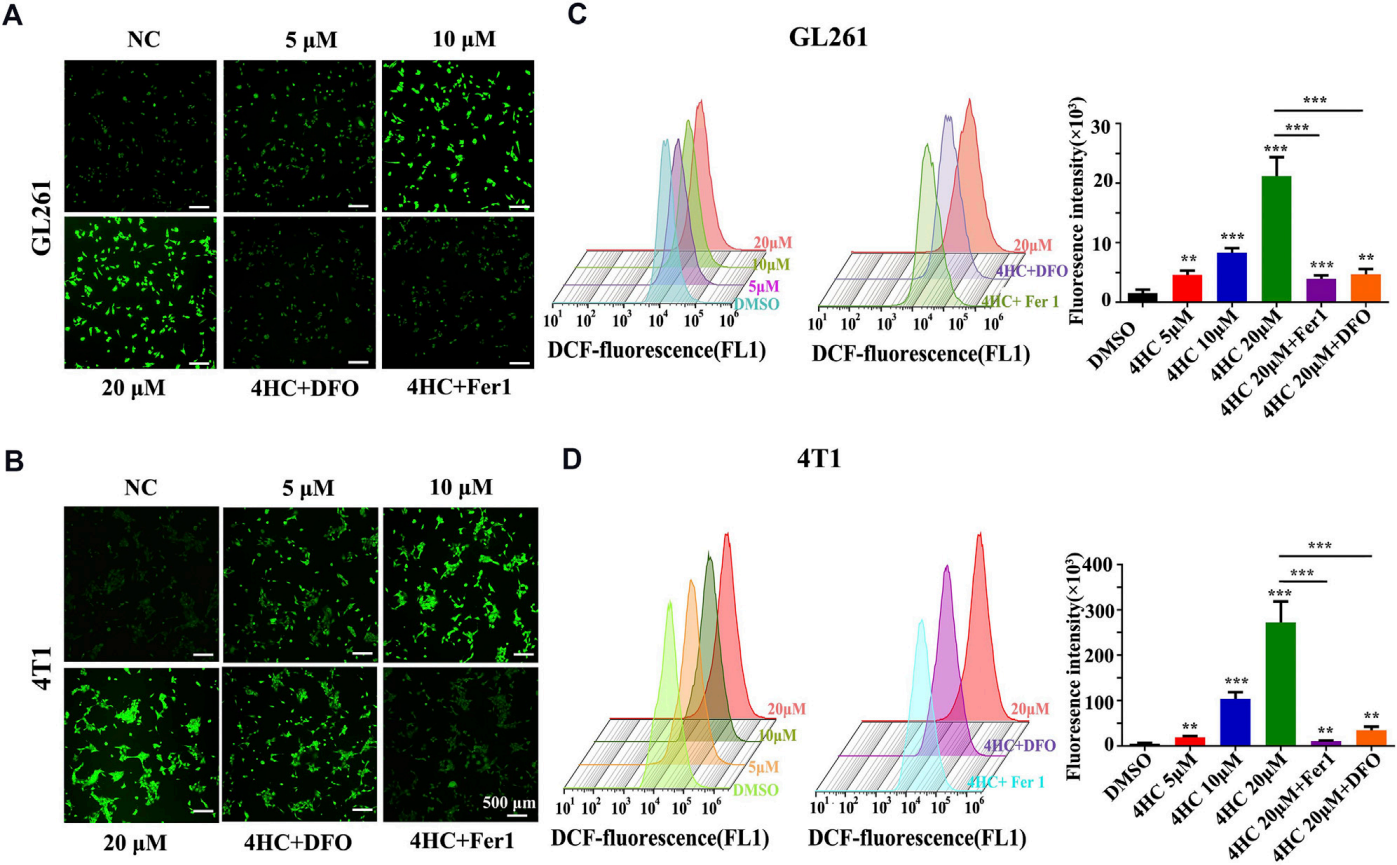
Ferroptosis inhibitors blocked 4HC-induced oxidative stress. Cultured GL261 and 4T1 cells were exposed to 4HC under the indicated conditions. **(A–B)** Representative images showing ROS levels in tumor cells using DFC. **(C–D)** Determination and quantification of the DFC fluorescence intensity using flow cytometry. Values are represented as the means ± SD from more than three independent experiments. ns, not significant, *p < 0.05, **p < 0.01, and ***p < 0.001 (one-way ANOVA analysis) for the indicated comparisons.

**FIGURE 4:**
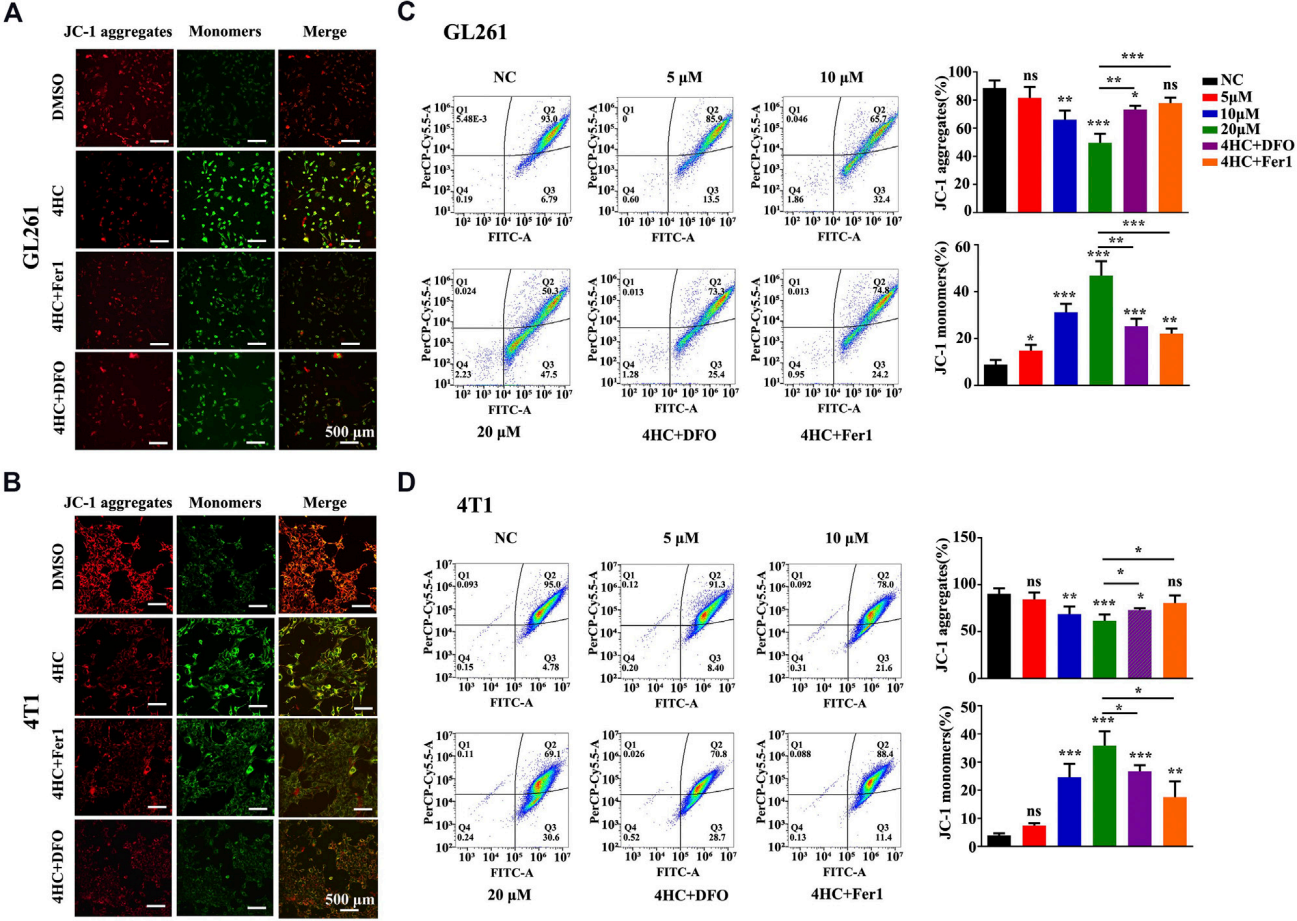
Ferroptosis inhibitors reversed the 4HC-induced decrease of the mitochondrial membrane potential. **(A–B)** Images of JC-1 staining in 4HC-treated GL261 and 4T1 cells cultured in the presence or absence of Fer1 and DFO. Shown were representative images with similar results (scale bar = 500 μm). **(C–D)** Determination and flow cytometry analysis of the MMP in tumor cells treated under the indicated conditions. Values are presented as the means ± SD from more than three independent experiments. ns, not significant, *p < 0.05, **p < 0.01, and ***p < 0.001 (one-way ANOVA analysis) for the indicated comparisons.

Based on the findings described above, we further confirmed the validity of ferroptosis as one of the main causes of CTX-induced cell death by applying various inhibitors of ferroptosis, such as ferrostatin-1 (Fer1) and deferoxamine (DFO). The viability of the GL261 and 4T1 cell lines treated with 4HC was significantly reduced in a dose- and time-dependent manner compared with that of the control groups, accompanied by increased cytotoxicity (Figures 2A,B). As shown in Figures 2C,D, 4HC treatment also altered the cell morphology under an inverted microscope, which was characterized by a reduced neurite-like shape and broadened intercellular gaps. Notably, 4HC-induced cell death and morphological changes were significantly attenuated in the presence of Fer1 or DFO (Figures 2C–E). Since ferroptosis depends on ROS production for its cytotoxicity (Dixon et al., 2012), we also observed prominent intracellular ROS accumulation in both GL261 and 4T1 cells (Figures 3A,B) after 4HC treatment, as visualized by the strong fluorescence intensity of the DCF probe. The quantitative flow cytometry analysis clearly showed the induction of oxidative stress in a concentration-dependent manner, which was almost completely offset by ferroptosis inhibitors (Figures 3C,D).

Furthermore, mitochondrial physiological function was evaluated by assessing the mitochondrial membrane potential (MMP) with JC-1 staining reagent in live cells. As shown in Figures 4A,B, 4HC reduced the MMP, as evidenced by a pronounced decrease in JC-1 aggregate formation (with specific red fluorescence), and more free green puncta indicating JC-1 monomers. However, pre-treatment with ferroptosis inhibitors (Fer1 or DFO) significantly restored the loss of the MMP induced by 4HC. Moreover, the quantitative flow cytometry analysis further indicated a concentration-dependent decrease in the MMP of 4HC-stimulated tumor cells (Figures 4C,D).

**FIGURE 5:**
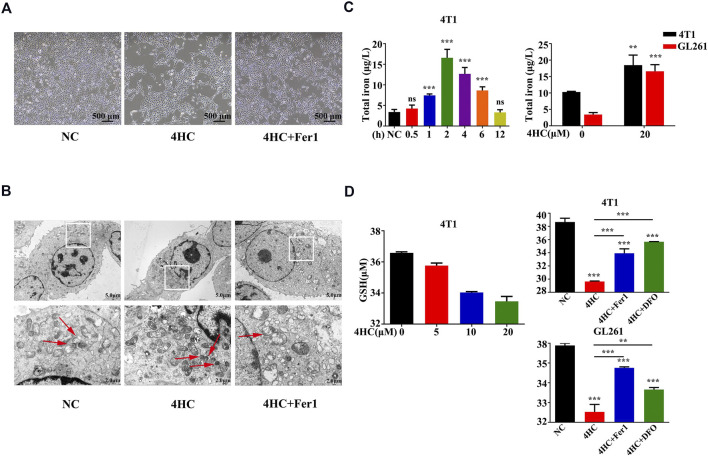
Mitochondrial deformation and 4HC-induced alterations in GSH or iron levels were antagonized by ferroptosis inhibitors **(A-B)** TEM visualization of changes in mitochondrial morphology in 4T1 cells exposed to various experimental conditions. The red arrows indicate mitochondrial. Shown were representative images with similar results (scale bar = 2.0 μm). **(C)** Intracellular iron concentrations in 4T1 and GL261 cells exposed to 4HC (20 μM) for the indicated times were determined using ICP–MS. Values are presented as the means ± SD from three independent experiments. ns, not significant, **p < 0.01, and ***p < 0.001 (one-way ANOVA analysis). **(D)** Intracellular GSH levels in 4T1 and GL261 cells exposed to the indicated conditions were determined. Values are represented as the means ± SD from more than three independent experiments. **p < 0.01 and ***p < 0.001 (one-way ANOVA analysis) for the indicated comparisons.

As one of the distinct morphological features of ferroptosis, mitochondria appear smaller than normal, with an increased membrane density and reduced cristae (Yagoda et al., 2007; Dixon et al., 2012). In our study, the transmission electron microscopy (TEM) analysis displayed similar mitochondrial deformation in 4HC-treated cells, which was blocked by Fer1 (Figures 5A,B).

Iron, a basic component within the mitochondrial respiratory chain, is required for ferroptosis (Dixon et al., 2012), (Gkouvatsos et al., 2012). We measured the intracellular iron concentrations and found that iron accumulated after treatment with 4HC and reached its highest level at the 2-h time point (Figure 5C). Glutathione (GSH) exhaustion is another critical event leading to excess oxidative stress during ferroptosis-mediated cell death (Gao et al., 2015; Yan et al., 2021); hence, we observed reduced GSH levels after 4HC treatment, and these changes were also inhibited by Fer1 or DFO (Figure 5D). All the results collectively demonstrate that ferroptosis is the relevant mechanism underlying the effects of CTX on inducing cell death.

### 3.3 HMOX-1 Played a Pivotal Role in the CTX-Induced Ferroptosis of Tumor Cells

**FIGURE 6:**
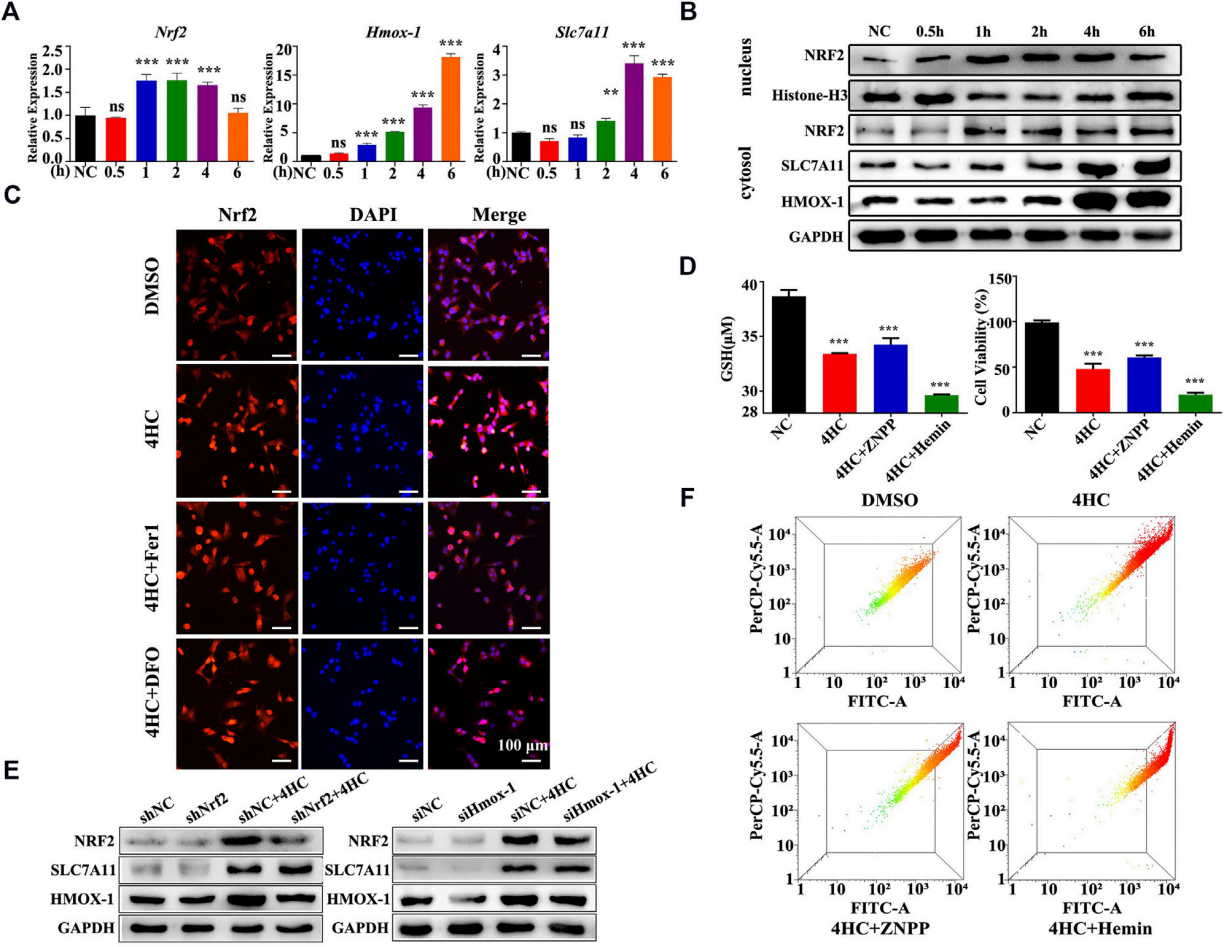
The NRF2/HMOX-1 pathway plays a role in CTX-induced ferroptosis. **(A)** Relative Nrf2, Hmox-1 and Slc7a11 mRNA levels in 4T1 cell lines treated with 4HC (20 μM) for the indicated times. Data are represented as the means ± SD from three independent experiments. ns, not significant, ^**^ *p* < 0.01, and ^***^ *p* < 0.001 (one-way ANOVA analysis). **(B)** Western blot analysis of NRF2/HMOX-1 system expression in 4HC-treated cells. Histone H3 served as a loading control, and representative results from three experiments are shown. **(C)** Images of immunofluorescence staining for NRF2. Representative images from experiments with similar results are shown (scale bar = 100 μm). **(D)** Intracellular GSH levels and the viability of cells after exposure to the specific HMOX-1 inhibitor zinc protoporphyrin (ZNPP) and agonist Hemin. Values are presented as the means ± SD from more than three independent experiments. ***p < 0.001 (one-way ANOVA analysis). **(E)** Western blot analysis of NRF2, HMOX-1 and SLC7A11 levels in transfected cells. GAPDH served as a loading control, and representative results from three experiments are shown. **(F)** Flow cytometry analysis of the MMP in cells treated under the indicated conditions; representative results from three experiments are shown.

HMOX-1 expression is regulated by nuclear factor E2 related factor 2 (NRF2), a main regulator of detoxifying/antioxidant phase II enzymes (Biswas et al., 2014; Chiang et al., 2018). Moreover, according to a previous study, increasing HMOX-1 expression increases the labile iron pool (LIP) and triggers ferroptosis (Chang et al., 2018). In this study, we logically hypothesized that the NRF2/HMOX-1 pathway plays a role in CTX-initiated ferroptosis and performed experiments to test our hypothesis. The expression of the Nrf2 mRNA was obviously upregulated after 4HC treatment for 1 h but returned to normal levels at 6 h (Figures 6A,B). Meanwhile, the nuclear translocation of NRF2 was also increased in the 4HC group, with stronger nuclear NRF2 expression observed using immunofluorescence staining (Figures 6B,C). To verify the up- and downstream regulatory mechanisms, cells were transfected with a small interfering RNA against Hmox-1 or short hairpin RNA against Nrf2. As expected, Nrf2 knockdown attenuated the levels of SLC7A11 and HMOX-1, but Hmox-1 silencing had no significant effect on the expression of NRF2 and SLC7A11 (Figure 6E), indicating that the NRF2/HMOX-1 signaling pathway was activated by the CTX treatment. Furthermore, we used a specific inhibitor or inducer of HMOX-1 to determine whether HMOX-1 is a key protein involved in CTX-induced ferroptosis. Compared with the 4HC group, treatment with the HMOX-1 inhibitor zinc protoporphyrin (ZNPP) noticeably reduced the cell viability and intracellular GSH level, as well as the MMP; in contrast, these ferroptosis-related features were more significantly increased in the HMOX-1 agonist (Hemin) group (Figures 6D,F). Therefore, HMOX-1 plays a pivotal role in promoting CTX-induced cancer cell ferroptosis.

### 3.4 The HMOX-1 Agonist Enhanced the Antitumor Effect of CTX by Promoting Ferroptosis *in vivo*

**FIGURE 7:**
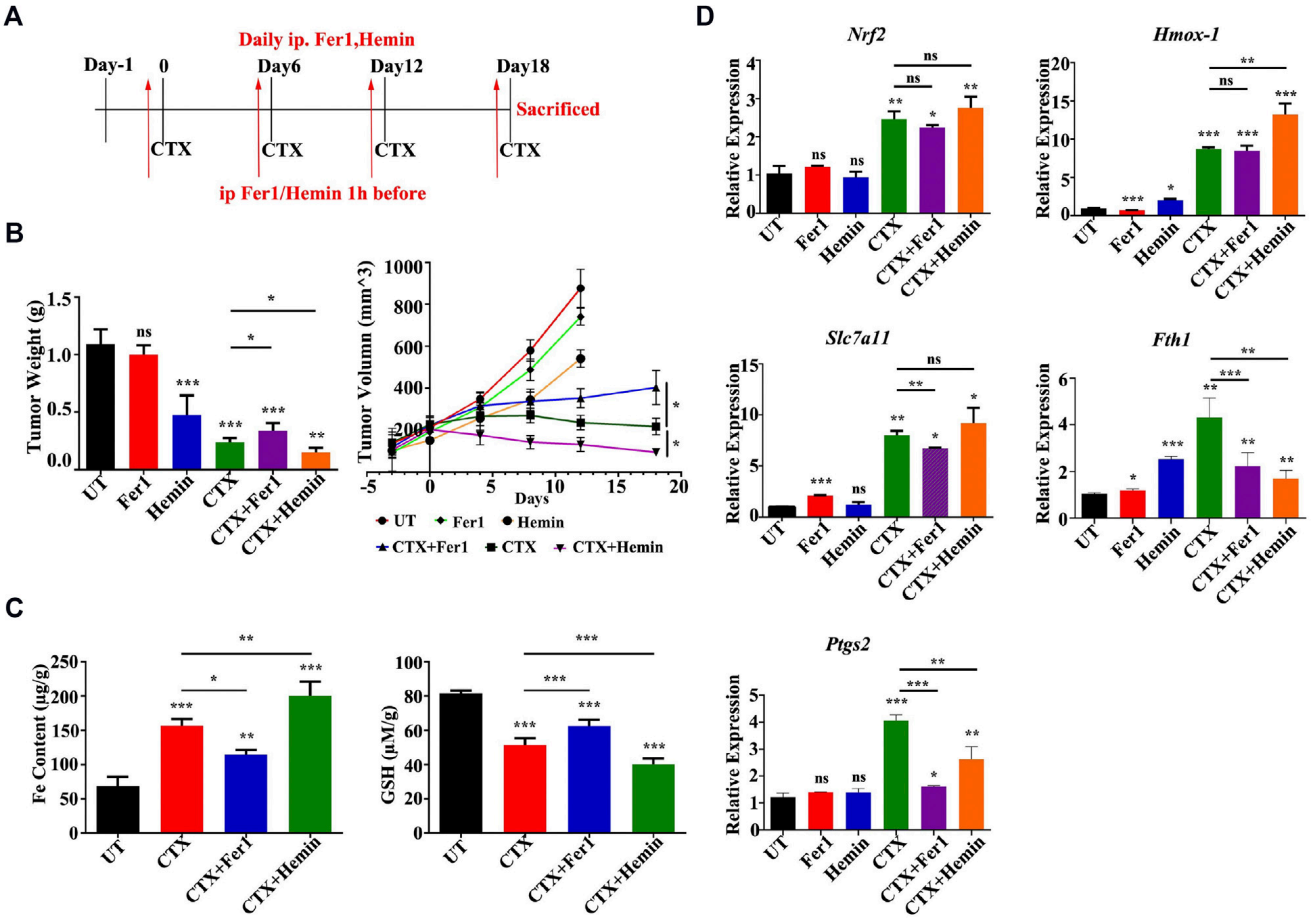
An HMOX-1 agonist enhanced the chemotherapeutic effect of CTX by inducing ferroptosis. **(A)** The murine xenograft model was established and treated as described in the text. **(B)** Tumor weight and volume of each group (*n* = 6–7) for the indicated times. Data are presented as the means ± SEM. ns, not significant, *p < 0.05, **p < 0.01, and ***p < 0.001 (one-way ANOVA analysis) for the indicated comparisons **(C)** Iron content and GSH levels in each group (*n* = 6–7). Data are presented as the means ± SEM. *p < 0.05, **p < 0.01, and ***p < 0.001 (one-way ANOVA analysis) for the indicated comparisons **(D)** Relative Nrf2, Hmox-1, Slc7a11, Fth1 and Ptgs2 mRNA levels in different groups (*n* = 6–7). Data are presented as the means ± SEM; ns, not significant, *p < 0.05, **p < 0.01, and ***p < 0.001 (one-way ANOVA analysis) for the indicated comparisons.

Based on the *in vitro* findings described above, a murine xenograft model of 4T1 breast cancer was established to examine the antitumor mechanism of CTX in BALB/c mice *in vivo*. 4T1 tumor-bearing mice were intraperitoneally injected with CTX (140 mg/kg) with or without Fer1 (4 mg/kg), Hemin (25 mg/mg) or the control (PBS) every 6 days (Figure 7A). CTX treatment significantly inhibited tumor growth in mice (Figure 7B), with more iron accumulation and decreased GSH level in tumor tissues (Figure 7C). Co-treatment with Fer1 abrogated these effects while treatment combined with HMOX-1 agonist Hemin enhanced these effects (Figures 7B,C). Consistently, the mRNA expression of Nrf-2, Hmox-1 and Slc7a11 were obviously upregulated in CTX group, as well as Fth1 and Ptgs2, two downstream key genes involved in ferroptosis (Figure 7D). However, compared with the CTX group, the CTX + Fer1 and the CTX + Hemin group did not show significant changes in Nrf2, Hmox-1 and Slc7a11 levels (Figure 7D). These results implied that CTX promoted tumor cell ferroptosis and combination treatment with HMOX-1 agonist could enhanced its chemotherapy effect in animals, which involved NRF2/HMOX-1 system.

## 4 Discussion

In this study, we first identified the classical chemotherapeutic drug CTX as a new ferroptosis inducer with a novel mechanism. The main mechanism by which CTX exerts its antitumor effects was to induce ferroptosis in cancer cells. We showed that HMOX-1 mediates CTX-induced ferroptosis by dysregulating cellular redox regulation. Furthermore, combination therapy with CTX and an HMOX-1 agonist significantly enhanced their antitumor activity. These findings indicated that ferroptosis has good potential as a new therapeutic intervention for cancer treatment.

Ferroptosis is generally referred to as a mode of programmed cell death involving the production of iron-dependent ROS that is distinct from other forms of cell death, based on morphological, biochemical, and genetic criteria (Dixon et al., 2012; Tang and Kroemer 2020). Ferroptotic cells exhibit completely different changes under the transmission electron microscope: abnormal, small mitochondria with reduced cristae and rupture of the outer membrane (Stockwell et al., 2020; Zhi et al., 2021). A novel tumor treatment strategy is to induce ferroptosis in tumor cells. Both chemotherapy and radiotherapy exert antitumor effects by inducing ferroptosis in tumor cells (Chen et al., 2021; Lei et al., 2021). Among various anticancer agents, CTX is a widely used anticancer agent for the treatment of solid tumors and hematological malignancies. CTX is an inactive prodrug activatesd by the hepatic cytochrome P-450 enzyme system to form 4HC (Emadi et al., 2009). *In vitro* experiments, CTX typically shows little or no activity, but its chemically activated derivative 4HC exhibits potent cytotoxic and immunomodulatory properties. Our observations are the first to show that CTX exerted its cytotoxic effects on different carcinoma cells by inducing ferroptosis. Indeed, activation of an alternative cell death pathway may provide a new opportunity to overcome resistance to chemotherapeutic agents. A strategy combining CTX as a ferroptosis inducer with other antitumor therapies for clinical treatment deserves further study.

Ferroptosis inducers (FINs) are divided into four categories based on different molecular mechanisms. Class I (Class I FINs) mainly inhibit the cystine/glutamate antiporter (System X_C_-) or glutamate-cysteine ligase (GCL) to consume intracellular glutathione (GSH) followed by ferroptosis (Hassannia et al., 2019). Guo J. et al. reported that classical platinum anticancer drugs, such as cisplatin, induce ferroptosis and that GSH depletion together with the inactivation of GPXs played a vital role in the underlying mechanism (Guo et al., 2018). Both Class II and class III FINs trigger ferroptosis by inhibiting GPX4 (Yang et al., 2014). Class II FINs directly inhibit GPX4 activity, while Class III FINs indirectly consume GPX4 through the mevalonate (SQS-mevalonate) pathway. Altretamine has been approved for the clinical treatment of ovarian cancer because it inhibits the activity of GPX4 to induce ferroptosis in ovarian cancer cells (Woo et al., 2015). The effects of Class I, II and III FINs are mediated by the classical pathway of ferroptosis induction because they all directly or indirectly inhibit GPX4 activity. Class IV FINs increase HMOX-1 levels to increase the labile iron pool (LIP), triggering ferroptosis, which is a nonclassical pathway. Our RNA-seq data showed that 4HC did not affect Gpx4 expression, but it increased Hmox-1 expression *in vitro*. Based on the effects on ROS generation, GSH depletion, iron accumulation, and compensatory upregulation of SLC7A11, CTX should be categorized as a Class IV ferroptosis inducer. Compared with normal cells, tumor cells require more iron for their metabolism and are more sensitive to ferroptosis induced by increased LIP (Pinnix et al., 2010; Li and Li 2020). Therefore, Class IV FINs are considered to have clinical application prospects.

Beyond the ferroptosis, some other enriched pathways related to endoplasmic reticulum and MAPKs were revealed by RNA-seq. CTX has been found associated with endoplasmic reticulum stress in multiple organs and diseases other than tumor (Mohammad et al., 2012; Long et al., 2015; Li et al., 2021). The MAPKs are proline targeted serine-threonine kinases, which are transducers of environmental stimulus to the nucleus. The p38-MAPK is a member of the MAPK family which is also called stress activated protein kinase pathways and often deregulated in cancers (Wagner and Nebreda 2009). Previous study found that CTX exerted its toxicity through cell apoptosis by activating p38 MAPK pathway in breast cancer cell line (Pang et al., 2011).

NRF2 is considered the central transcriptional regulator of redox stress in cells (Ma 2013; Furfaro et al., 2016). Accumulating data suggest that oxidative stress is a trait of kidney damage, cardiotoxicity, liver injury, and myelosuppression induced by CTX administration (Emadi et al., 2009; Ponticelli et al., 2018; Ayza et al., 2020). Upon activation, NRF2 translocates from the cytoplasm to the nucleus and interacts with AREs to induce the expression of downstream antioxidant genes, such as HMOX-1. However, contradictory results for the role of the NRF2/HMOX-1 pathway in ferroptosis have been reported. Sun X et al. documented a central role for the NRF2/HMOX-1 pathway in protecting hepatocellular carcinoma cells from ferroptosis since Hmox-1 knockdown enhanced cell growth inhibition induced by erastin (one of the ferroptosis inducers) and sorafenib (Sun et al., 2016). A similar result for the ability of HMOX-1 to attenuate ferroptosis induction was also observed in renal proximal tubule cells (Adedoyin et al., 2018). In contrast to its negative role in ferroptosis, several studies have shown that increased HMOX-1 expression augments or mediates anticancer agent (Bay117085 and withaferin A)-induced ferroptosis by promoting iron accumulation and ROS production (Chang et al., 2018; Hassannia et al., 2018). In our study, gene upregulation of Hmox-1 and Slc7a11 were observed within 6 h after CTX treatment, and CTX caused a transient increase in NRF2 translocation to the nucleus, followed by a dramatic increase of HMOX-1 expression, leading to mitochondrial dysfunction and ferroptosis-mediated cell death. Therefore, we speculated that CTX might induce ferroptosis through NRF2/HMOX-1 system in an very early stage, and then exert cytotoxic effects by affecting DNA synthesis and other mechanisms. Chiang SK et al. postulated that HMOX-1 is activated as a cytoprotective defense mechanism or governs ferroptotic progression that depends on the degree to which HMOX-1 expression is increased in response to stimulatory cues (Chiang et al., 2018). When HMOX-1 expression is moderately activated, NRF2-derived HMOX-1 exerts a cytoprotective effect by neutralizing ROS; while a high degree of HMOX-1 activation increases LIP, leading to ROS overload and subsequent oxidative cell death (Chiang et al., 2021). Our *in vivo* and *in vitro* experimental results using the HMOX-1 agonist Hemin support this hypothesis, and the application of HMOX-1 modulators to mediate ferroptosis might be a new strategy for cancer chemotherapy.

In conclusion, the present study is the first to show that CTX induces ferroptosis and HMOX-1-mediated redox regulation plays a vital role in the underlying mechanism. Based on these results, ferroptosis has great potential as a new approach in antitumor therapies and provides a new opportunity to use classic chemotherapy agents.

## Data Availability

The data presented in the study are deposited to the repository (the SRA database accession number PRJNA799495).
